# Wild Avian Gut Microbiome at a Small Spatial Scale: A Study from a Mediterranean Island Population of *Alectoris rufa*

**DOI:** 10.3390/ani13213341

**Published:** 2023-10-27

**Authors:** Monica Guerrini, Dalia Tanini, Claudia Vannini, Filippo Barbanera

**Affiliations:** Department of Biology, University of Pisa, Via A. Volta 4, 56126 Pisa, Italyfilippo.barbanera@unipi.it (F.B.)

**Keywords:** host-microbiome associations, microbiome, non-invasive sampling, red-legged partridge, 16S rRNA metabarcoding

## Abstract

**Simple Summary:**

Our study is one of the few comparative and within-a-species descriptions of microbiomes in wild non-passerine birds. Particularly, it focuses on red-legged partridges, which are medium-sized gamebirds inhabiting open dry countryside and low-intensity cultivations with a mix of fallow and uncultivated areas in southwestern Europe. We wanted to study microbes living in their gut as their occurrence and diversity may affect both survival and reproduction of these birds. We collected fresh red-legged partridge fecal pellets at different sites located on both the western (two) and eastern (one) sides of Elba Island (central Italy). Although most represented bacteria were the same in all the three sites, we found differences between western and eastern Elban subpopulations in terms of microbiome composition and diversity. This result might be related to locally diverging individual physiological needs and/or to different intensities in past releases of captive-bred birds between the two sides of Elba. Overall, we suggest that the two partridge subpopulations should be managed separately to avoid any loss or significant variation in their microbiome structure.

**Abstract:**

This research is one of the few comparative descriptions at an intraspecific level of wild non-passerine microbiomes. We investigated for the first time the gut microbiome of red-legged partridges (*Alectoris rufa*) using fecal pellets in order to provide a more informed management. We focused on a small Italian population consisting of two demes (WEST, EAST) separated by about 20 km on the opposite sides of Elba Island. Given the small spatial scale, we set up a sampling protocol to minimize contamination from environmental bacteria, as well as differences due to variations in—among others—habitat, season, and age of feces, that could possibly affect the investigation of the three Elban sites. We found a significant divergence between the WEST and EAST Elban subpopulations in terms of microbial composition and alpha diversity. Although most represented bacterial phyla were the same in all the sites (*Firmicutes*, *Actinobacteria*, *Proteobacteria*, and *Bacteroidetes*), microbiomes displayed a much higher diversity in western than in eastern partridges. This result might be related to locally diverging individual physiological needs and/or to different intensities in past releases of captive-bred birds between the two sides of Elba. We suggest that the two subpopulations should be treated as distinct management units.

## 1. Introduction

The rapid development in cultivation-independent high-throughput sequencing techniques allowed the outbreak of ground-breaking research on what Woese [1] referred to as the ‘sleeping giant’ of biology: the microbial world. This process revealed the largely unforeseen role that microorganisms play in development, growth, and health of virtually all living beings since they harbor the so-called microbiome [2].

Among the vertebrates, it is well known that the microbiome of the gastrointestinal tract can affect survival and reproductive performance through its interactions not only with nutrition but also with both the physiology and immune system of the host [3,4,5]. Symbiotic microbes can indeed play a pivotal role in herbivores’ digestion [6,7], in fulfilling specific nutritional requirements (e.g., in whales [8]), and in protection against pathogens (e.g., [9,10]). Perturbations or even disruption of microbial communities—most commonly of the gastrointestinal tract, the so-called dysbiosis (sensu [11])—are often associated with a health disorder and can potentially result in a significant decline of both survival rate and fitness of the host [12,13]. Despite its role in nutritional uptake, detoxification, immune function, and competitive exclusion of pathogens [14,15], the gut microbiome has been less explored in wild birds than in mammals [16], with the non-passerine species being studied very rarely [17,18]. Therefore, a better understanding of wild bird microbiomes necessarily implies widening the range of investigated taxa.

**Figure 1:**
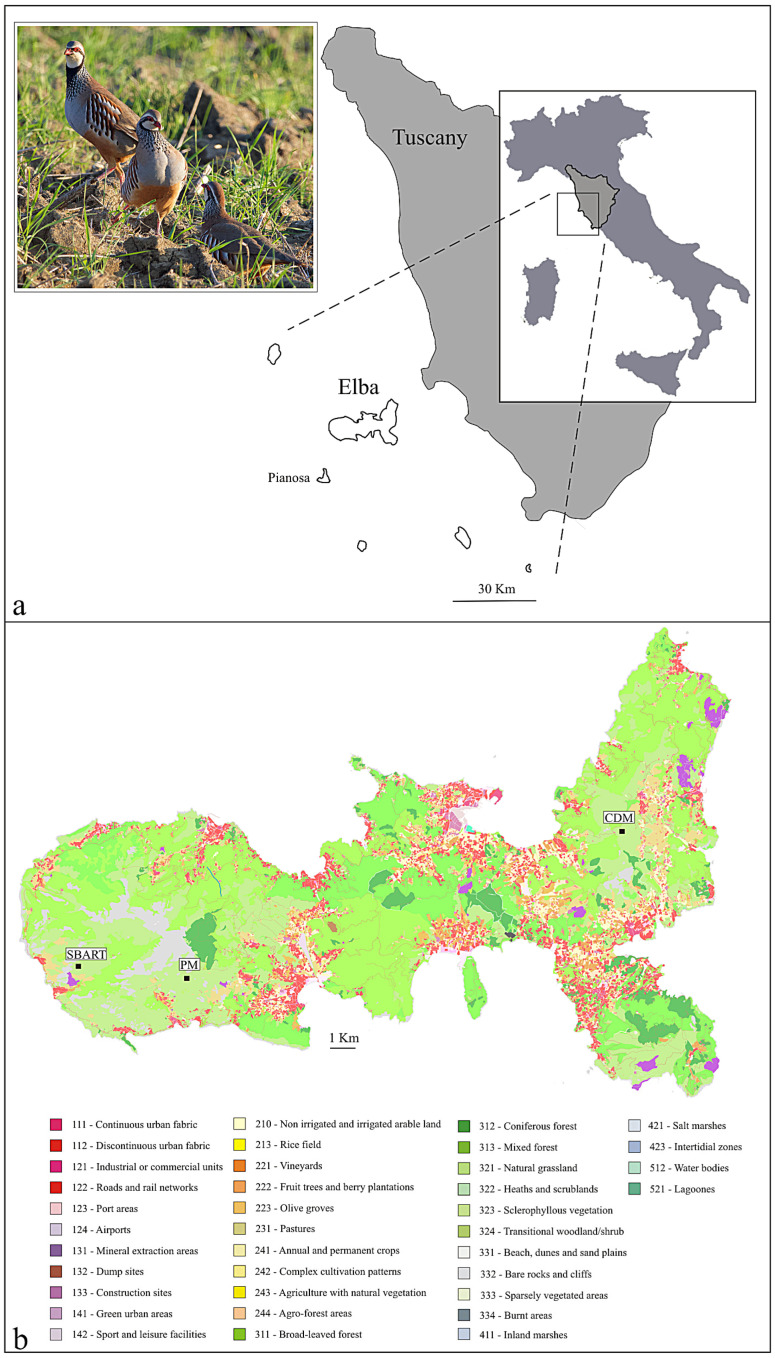
(**a**) The position of the island of Elba in Tuscany, central Italy; inset: a few red-legged partridges in the wild (courtesy: J.J. Negro, Spain). (**b**) Land cover map of Elba (source: Tuscan Region GEOscopio WMS: UCS10k 2019) obtained using QGIS v. 3.6 ‘Noosa’. The three sampling localities (same land class, n. 323) are indicated: SBART, San Bartolomeo; PM, Pietra Murata; CDM, Cima del Monte (see also Table 1).

The red-legged partridge (*Alectoris rufa*, Phasianidae) is a medium-sized gamebird that inhabits open dry countryside and low-intensity cultivations with a mix of fallow and uncultivated areas. In mainland Europe, it occurs from the Iberian Peninsula across most of France to northwestern Italy. Three subspecies are recognized: *A. r. rufa*, native to France and Italy, and *A. r. hispanica* and *A. r. intercedens* from northwestern Spain and the remainder of the Iberian Peninsula, respectively. Nominated subspecies have also been historically introduced into several islands, for instance, Corsica (VI c., [19]), Great Britain (XVII c., [20]), and—to the easternmost edge of its range—Elba, in the Tuscan Archipelago (Figure 1a). This latter consists of seven main islands, with Elba—the third in Italy by size (223.5 km^2^)—hosting a small, protected red-legged partridge population, which is the only natural (not from an ex situ facility), long-established (at least since the late 1700s but likely much earlier [21]), and self-sustaining (no supplementation since the mid-1990s, [22]) Italian resource of this species. Nonetheless, partridges have long been hunted on Elba and despite the establishment of the Tuscan Archipelago National Park (1996)—with the majority of the island territory set under strict protection—a demographic collapse eventually took place by the end of 1990s, when the occurrence of only 30–50 pairs was assessed (see [21] and references therein). Later, Chiatante and colleagues [23] could not estimate any reliable population density value due to the paucity of individuals.

In a previous study based on the use of microsatellite DNA markers [22], we assessed that western and eastern regions of the Island, which are separated by less than 20 km, host genetically diverging subpopulations. In this study, we collected samples from western (two) and eastern (one) sites of Elba Island to compare microbial communities associated with the local red-legged partridges. On the one hand, we wanted to provide one of the few comparative descriptions at an intraspecific level of wild non-passerine bird microbiomes. On the other hand, we sought to gain first microbial data that would be useful to improve the management of either wild or farmed *A. rufa* using the Elban population as a reference.

## 2. Materials and Methods

### 2.1. Biological Sampling

We selected three study sites within the limits of the National Park: two in the west (San Bartolomeo, 42°45′23.35″ lat. N and 10°07′31.56″ long. E, SBART; Pietra Murata, 42°45′15.03″ lat. N and 10°11′01.67″ long. E, PM) and one in the east (Cima del Monte, 42°47′51.74″ lat. N and 10°23′27.95″ long. E, CDM) Elba (Table 1). Located at a similar elevation (SBART, 402 m a.s.l.; PM, 547 m; CDM, 428 m) and holding the same exposure to the sun (open 360° view), these sites were assigned to the same land class (Figure 1b), which is dominated by herbaceous plants with garrigue and sclerophyllous vegetation among small grassland patches. They are largely characterized by *Brachypodium retusum* (Poaceae) and *Foeniculum vulgare* (Apiaceae), with *Phagnalon saxatile* (Asteraceae) and *Micromeria graeca* (Lamiaceae) occurring mainly in the CDM site. The lower herbaceous layer and the open spaces with meadows include *Poa annua* (Poaceae), *Trifolium subterraneum* (Fabaceae), *Hypochaeris achyrophorus* (Asteraceae), *Polycarpon tetraphyllum* (Caryophyllaceae), and *Plantago bellardii* (Plantaginaceae), whereas *Lavandula stoechas* (Lamiaceae) and *Cistus monspeliensis* (Cistaceae) are mainly found in the bushy areas, with *C. salviifolius* being more abundant on the western side of Elba [24,25]. Fresh (no more than 2–3 h old, to minimize contamination from environmental bacteria) and well-spaced fecal pellets were individually collected in winter (SBART, February 2019; PM, February 2020; CDM, December 2018) at least three days after the last rainfall, from 8.00 to 10.00 a.m., and with an air temperature ranging −2 to 4 °C. Separately kept in plastic vials (no chemicals added), samples were transported according to a strict cold chain until the final storage (−40 °C) at the University of Pisa. Six pellets for each site were randomly selected and used for the analyses (total sample size, 18).

### 2.2. DNA Extraction

Sample manipulations and DNA extractions were carried out under a dedicated sterile cabinet (Polaris 48, Steril Spa) and all materials and disposables were sterilized with UV light for 2 h. After the removal of a white layer (urine) that can be typically found on the top, genomic DNA was extracted using 200 mg of feces from a single pellet and the QIAamp^®^ Fast DNA Stool Mini Kit (Qiagen, Hilden, Germany) according to the manufacturer’s instructions with minor changes. We added 1 mL of InhibitEX^®^ buffer, then we heated 5 min at 95 °C to improve the lysis, and the final elution was in 120 μL of Buffer ATE. A blank extraction (no sample) was included in each session. DNA concentration was assessed with a Qubit 2.0 fluorometer and Qubit dsDNA HS Assay Kit (Thermo Fisher Scientific, Waltham, MA, USA).

### 2.3. 16S rRNA Gene Amplification and Sequencing

Amplifications were carried out under a different sterile cabinet (Top Safe 1.2, BioAir), with all materials, disposables, and the surface sterilized with UV light for 2 h before the setup of reactions. The V3–V4 regions of the 16S rRNA gene were amplified using the (5′-3′) primers (forward) 341F-CCTACGGGNGGCWGCAG and (reverse) 785R-GACTACHVGGGTATCTAATCC of [26]. The Illumina overhang adapter sequences added to the forward and reverse primers were TCGTCGGCAGCGTC AGATGTGTATAAGAGACAG and GTCTCGTG GGCTCGGAGATGTGTA TAAGAGACAG, respectively. Each PCR was performed in 50 µL using the KAPA HiFi HotStart Ready Mix (Roche Diagnostics, Pleasanton, CA, USA), 8 ng of DNA, 0.2 µM of each primer, and adding 0.3 µg/µL of Ultrapure BSA (Bovine Serum Albumin, Invitrogen, Waltham, MA, USA). The thermal profile comprised 5 min at 95 °C, followed by 40 cycles of 30 s at 95 °C, 30 s at 55 °C, and 30 s at 72 °C, and a final extension at 72 °C for 5 min. The concentration of the amplicons was quantified using a Qubit fluorometer. Then, amplicons were barcoded and a sequencing library from each sample with an average concentration of 27.38 ng/µL was obtained. All 18 amplicons were sequenced on the Illumina MiSeq platform (sequencing depth of 2 × 100,000 paired-end reads) by IGATech (Udine, Italy).

### 2.4. Analysis of the Sequences

Raw reads of prokaryotic V3–V4 regions were analyzed using the Quantitative Insights Into Microbial Ecology version 2 (Qiime2, https://qiime2.org, accessed on 1 March 2023) software package [27]. Reads were truncated at 260 bp length to remove the lower-quality last base calls. After that, quality filtering, primer trimming, pair-end read merging, and de novo chimera removal were performed with the divisive amplicon denoising algorithm (Dada2) plugin [28]. The resulting sequences were then used to generate amplicon sequence variants (ASVs). ASVs displaying a total abundance lower than 10 were discarded before proceeding with downstream analyses. ASV sequences were aligned with Mafft [29] and a phylogenetic tree was inferred with FastTree [30]. This latter was manually inspected and no further chimeric sequence was disclosed. Taxonomic assignment of sequence variants was carried out using the release 132 of the Silva database [31]. A Naive Bayes classifier was trained extracting the regions of interest from SSU rRNA representative sequences (99% similarity clustered Operational Taxonomic Unit) as in [32]. Sequence variants identified as mitochondria, chloroplasts, unassigned, as well as all non-bacteria were removed before further data processing. 

### 2.5. Statistical Analyses

All statistical analyses were performed using Qiime2 either for the three populations (SBART, PM, and CDM) and the western (WEST) and eastern (EAST) subpopulation. Taxa bar plots were produced at the phylum and genus level. Alpha and beta diversity were then estimated using ASVs. Rarefaction curves for each individual were obtained with a depth of 7701. Alpha diversity was assessed by calculating three different indexes: number of sequence variants (ASVs), Shannon’s index (quantitative, non-phylogeny based index) for richness, and Pielou’s Evenness for evenness. Comparison among index values for different communities was performed by the Kruskal–Wallis non-parametric test. Different metrics—Bray–Curtis and Jaccard for quantitative and qualitative data, respectively, and both weighted and unweighted Uni-Frac distances to assess the impact of phylogeny—were used for calculating beta diversity by means of a multivariate Principal Coordinates Analysis (PCoA) and Permanova (pairwise comparisons, 999 permutations).

## 3. Results

### 3.1. Sequencing Outcome

High-quality 16S rRNA gene sequences (2,092,950) were obtained from 18 fecal samples. The number of reads ranged 61,270–187,344 (116,275 ± 32,324, on average). After filtering all non-bacterial and unassigned sequences, the final dataset comprised 1,386,402 high-quality reads (77,022 ± 20,804, on average). The rarefaction curves reported in Figure S1 reached a plateau, thus confirming that the sequencing depth was sufficient to sample all variants in the libraries.

### 3.2. Composition of the Microbial Communities

**Figure 2:**
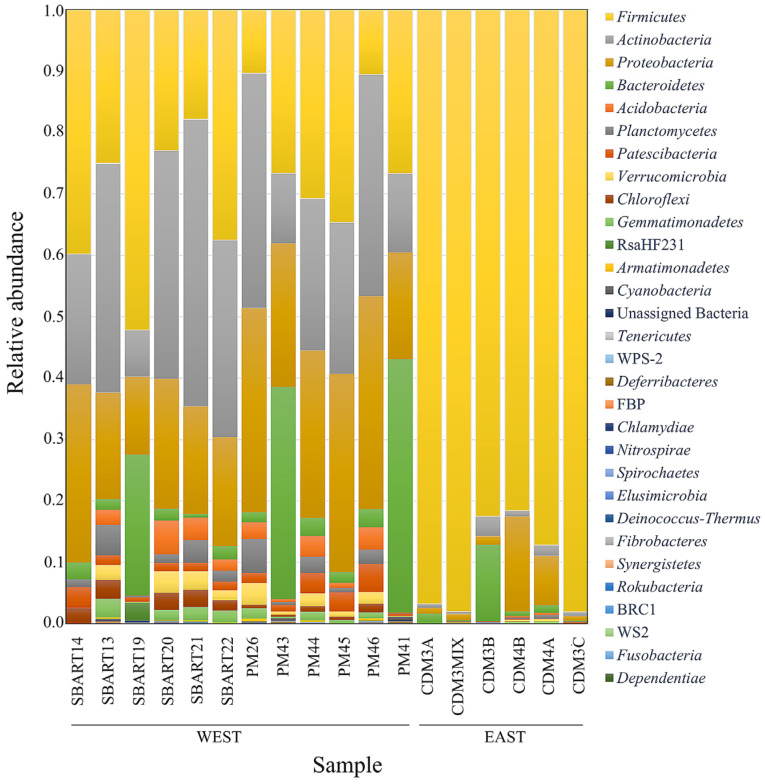
Relative abundances of bacterial phyla in the libraries as obtained for the three sampling sites. Each bar corresponds to one sample (single red-legged partridge).

The composition of the gut microbiome was reported for each partridge from the three study sites in the bar plots provided in Figure 2, in which differences between the WEST and EAST subpopulations concerning both the number of phyla and their relative abundance are visible. The western partridges (SBART and PM) showed a higher number of phyla and a more homogeneous distribution of the most represented ones (*Firmicutes*, *Actinobacteria*, *Proteobacteria*, and *Bacteroidetes*). On the other hand, *Firmicutes* was dominant in the EAST subpopulation with a relative abundance ranging 81–98% (Figure 2). The structure of the microbiome did not change when the relative abundance of taxa was investigated at the level of genus (Figure S2). The western partridges were characterized by a higher number and/or a more homogeneous distribution of taxa compared to the EAST subpopulation. In this, we found a lower number of genera along with a larger relative abundance of one (e.g., *Lactobacillus* in three out of six CDM samples) or of very few taxa than in the two western sites.

### 3.3. Comparative Analyses of Microbial Diversity

**Figure 3:**
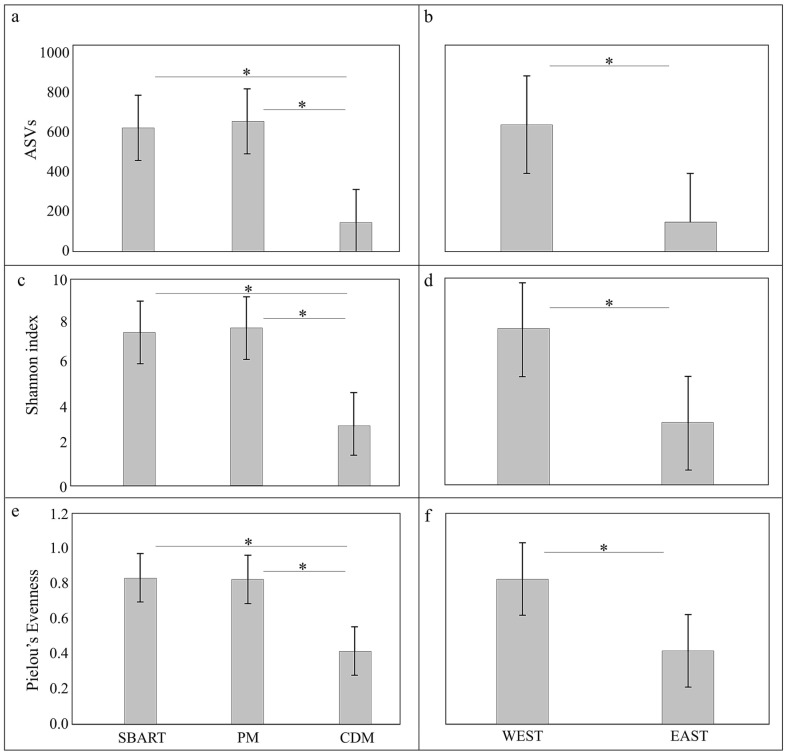
Measures of alpha diversity for the three sampling sites (SBART, PM, and CDM) and for each subpopulation (WEST and EAST) are reported as number of sequence variants, ASVs (**a**,**b**), Shannon (**c**,**d**), and Pielou’s Evenness indexes (**e**,**f**). Error bars indicate the Standard Error (SE). Results of Kruskal–Wallis tests are reported in Table S1. Statistically significant comparisons (*p* < 0.05) are reported with an asterisk (*).

Comparative estimates of alpha diversity (number of ASVs, Shannon, and Evenness indexes) for the three sites (SBART, PM, and CDM) and for the two subpopulations (WEST, EAST) were reported in Figure 3. The total number of ASVs was 3743, 3935, and 889 for SBART, PM, and CDM sites, respectively. The average number of ASVs was four times lower in CDM (148.17 ± 85.83) than in the other two localities (SBART, 623.83 ± 341.63; PM, 655.83 ± 216.47) (Figure 3). As far as the other two indexes are concerned, the average values obtained for SBART and PM were double than those of CDM (Shannon: SBART, 7.46 ± 1.10; PM, 7.67 ± 0.83; CDM, 3.03 ± 1.49; Pielou’s Evenness: SBART 0.84 ± 0.06; PM, 0.83 ± 0.07; CDM, 0.42 ± 0.16; Figure 3). The Kruskal–Wallis tests (Table S1) returned highly significant differences (*p* < 0.01) for all alpha diversity indexes between each of the western sites (SBART and PM) and the eastern one (CDM) but not between SBART and PM within the WEST subpopulation (*p* > 0.05: Table S1). Likewise, the Kruskal–Wallis test indicated the occurrence of highly significant differences between WEST and EAST for all indexes (*p* < 0.01; Table S1).

**Figure 4:**
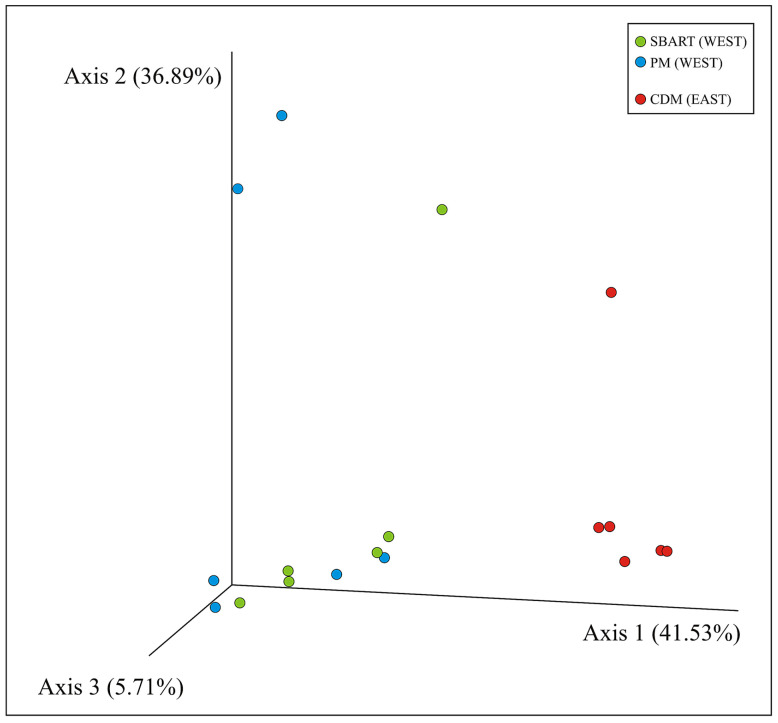
Principal Coordinates Analysis of microbial communities computed using weighted Uni-Frac distances and all available samples. Legend: green, SBART; blue, PM; red, CDM.

The PCoA of microbial communities as computed for all individuals using weighted Uni-Frac distances was reported in Figure 4 (similar results were obtained with Bray–Curtis, Jaccard, and unweighted Uni-Frac). Axes 1, 2, and 3 explained 84.13% of the total variability and a separation between the microbiomes from CDM and those from the two sites on the western side of Elba was disclosed. Permanova tests carried out for SBART, PM, CDM as well as for the two subpopulations (WEST, EAST) were reported in Table S2. All comparisons were statistically significant (*p* < 0.01) when Jaccard and Bray–Curtis distances were used—the two western sampling sites (SBART versus PM: *p* = 0.011) included—whereas with both weighted and unweighted Uni-Frac distances, SBART and PM were the only two sites that did not significantly diverge from each other (*p* = 0.206).

## 4. Discussion

Environmental conditions and diet are among the main factors shaping gut microbiome in wild passerine [33,34] and non-passerine [17,18] birds as well as in mammals. As to these latter, variation in the microbiome composition in wild populations, for instance, can be significantly driven by seasonal shifts in the diet [35,36,37]. Therefore, in the present work, consistency of habitat, foraging sources, and season among the sampling sites was deemed as a priority to mitigate the effects of exogenous factors. We investigated for the first time the gut microbiome of a non-model avian species: the red-legged partridge. According to the study of Turjeman and colleagues [38] in wild common cranes (*Grus grus*), non-invasive samples can even better represent host fecal microbial matter than those obtained from invasive ones as, for instance, there are no effects on the physiology of individuals due to the trapping. Hence, we used fecal pellets collected in localities within areas historically inhabited by *A. rufa* on the opposite sides of Elba Island [39] in herbaceous habitats—the preferred ones by partridges—that were highly consistent in terms of species’ assembly (see Materials and Methods). Also, sites held a similar elevation, with this being another environmental parameter potentially influencing gut microbiome composition [40] (Table 1). Whereas partridges feed mainly—but not exclusively—on insects in the early stages of their life to meet protein requirements, they become increasingly herbivorous as soon as they become adults at 1 year of age [41]. The sampling was carried out in winter; hence, we most likely sampled adult partridges—after post-breeding dispersal—feeding mainly on fruits and seeds and secondarily on herbaceous plants (e.g., leathery leaves from evergreen species). Therefore, given the alleged age of host individuals and the high similarity in plant coverage (see Materials and Methods and Figure 1) of the sampled habitats, we made our very best to ensure the highest possible consistency in accordance with the aims of the work, which did not include the analysis of the diet of birds (see also the last part of Discussion). Considering another aspect, the fecal microbiome of birds is also known to be contributed to by different segments of the gastrointestinal tract. For instance, the cecum turned out to be one of the most important sources in the Japanese quail (*Coturnix japonica*, [42]). Although the domestic chicken (*Gallus gallus*) can expel the cecum content two or three times per day, there is little temporal continuity in the fecal microbiome growth and very poor is known about the involvement of other gastrointestinal regions in this as well as in other avian species [43]. Furthermore, the fecal microbial community is also dynamic over time once released in the field [44]. Therefore, we selected a strict time frame (8.00–10.00 a.m.) for the sampling at each site, so as to standardize any possible influence of different gastrointestinal tracts, and we collected only fresh pellets, namely those defecated no more than 2–3 h before—according to the experience of one of us (F.B.)—to minimize any possible contamination. Overall, this protocol represents a promising avenue to set up future similar investigations as comparative studies are important to provide knowledge of mechanisms affecting host–microbe relationships in the wild [45].

We reported microbiome composition and spatial structure in a wild *A. rufa* island population. We identified either conserved bacterial phyla or differences between subpopulations in terms of the microbial community across the Elban territory. The most common phyla were *Firmicutes* (53.8%), *Actinobacteria* (16.3%), *Proteobacteria* (15.1%), and *Bacteroidetes* (8.1%), which is a result in agreement with [16,33], where these four taxa were referred to as the ‘core microbiome’ of wild avian species, as well as with [46], where non-passerine captive birds were investigated. Within Phasianidae, *Firmicutes*, *Bacteroidetes*, and *Proteobacteria*—with *Tenericutes* as the fourth taxon in order of abundance—were the most represented phyla occurring in the microbial community of farmed Japanese quails [42]. Likewise, *Actinobacteria*, *Firmicutes*, *Proteobacteria*, and *Synergistetes* were the main microbial groups identified in wild ptarmigans (*Lagopus muta*, [18]). Overall, the composition of the Elban *A. rufa* microbiome, having *Firmicutes*, *Actinobacteria*, *Proteobacteria*, and *Bacteroidetes* as dominant phyla, was strongly consistent with the previous findings.

*Firmicutes* are Gram-positive bacteria producing short-chain fatty acids (butyrate) as byproducts of fermentation that can be directly absorbed by the host as a source of energy. Interestingly, they were very abundant in the CDM subpopulation, with *Lactobacillaceae* and *Erysipelotrichaceae* as the most widespread families. On the one hand, *Lactobacillus salivarius* can be used as a probiotic in the diet of captive-bred birds to improve nutrient absorption and increase growth performance (in domestic chickens and ducks, [47,48,49]). On the other hand, *Erysipelotrichaceae* are plentiful in domestic chickens converting feed to mass in an efficient manner [50]. Although it may be plausible that divergent physiological needs can account for the difference in the abundance of *Firmicutes* between EAST and WEST subpopulations, richness in lactobacilli might be (also) related to massive restocking with captive-bred individuals carried out on Elba especially between the 1960s and mid-1990s, with a higher intensity on the eastern than on the western side of the island [21,39]. This consideration might also explain the lower degree of microbiome diversity observed in the eastern subpopulation, as lots of evidence indicates that variability is usually lower in ex situ than in situ facilities. Nevertheless, this pattern is not straightforward as there are numerous study cases where the occurrence of no significant difference between captive and wild individuals was recorded as well, with even a few ones—focusing also on mammals and birds—where captive populations beneficiated instead from a higher microbiome diversity than wild ones [51]. Although the microbiome of farmed individuals usually changes in a short time after their release in the wild (e.g., [52,53,54]), we cannot exclude that some adaptation in the gut microbiome of EAST partridges may have been selected in captive-bred birds of various geographical origin—also imported from abroad [39]—during intense restocking. It is worth noting, indeed, that the eastern side of Elba is characterized by a dry Thermo-Mediterranean bioclimate, whereas a superior Meso-Mediterranean—shifting to humid Supra-Mediterranean—occurs on the western side, where less garrigue and rocky outcrops and more wooded patches can be comparatively found [22]. Unfortunately, birds employed for supplementation on Elba were obtained from farms that no longer exist today [39]; hence, there is no chance to carry out any ad hoc comparative study of captive versus wild bird microbiomes.

Despite the small spatial scale, a significant divergence was found between western (SBART and PM) and eastern (CDM) Elban *A. rufa* subpopulations, as shown by taxonomic assemblages as well as both alpha and beta diversity analyses. Partridges from SBART and PM sites displayed a higher number of taxa and a more homogeneous distribution of their relative dominances compared to those from CDM. Indeed, values of all alpha diversity indexes in the WEST sites were significantly higher than those recorded in the EAST subpopulation (Figure 2 and Figure 3; Table S1). On the contrary, microbiomes of partridges sampled in SBART and PM sites accounted for a large homogeneity within the WEST subpopulation. This was also certified by Permanova tests and PCoA (Figure 4 and Table S2), thus pointing to the occurrence of distinct local adaptations by the two Elban *A. rufa* subpopulations.

The gut microbial communities turned out to be more closely related to each subpopulation (WEST, EAST: *c*. 20 km away one another) than to the sampling sites (SBART and PM within WEST). Interestingly, the same overall pattern was inferred by genotyping *A. rufa* partridges at a panel of 11 loci of the microsatellite DNA [22]. In this latter study, we investigated the partition of the genetic diversity among and within western and eastern Elban subpopulations and the results pointed to the occurrence of a well-established *A. rufa* spatial structure across the island. We found that the Elban population actually consisted of two demographically largely independent and genetically diverging groups resident in the opposite sides of the island. Overall, nuclear DNA diversity was high in the whole population—see also the lack of runs of homozygosity as inferred by [55]—and no significant difference in terms of allelic richness, Index of Nei, and observed heterozygosis was detected between the two subpopulations [22]. However, conventional wisdom suggests that the more the variables are controlled, the higher accuracy and precision of the survey will result. In the present study, we tried our very best to investigate the microbiomes of wild partridges in comparable conditions across the different sampling sites. However, an analysis of the diet of the sampled individuals (e.g., by mitochondrial metabarcoding of fecal pellets) was beyond the purposes of this study; hence, we are aware that we cannot assume that environmental factors were fully equivalent. Likewise, we cannot exclude that individual physiology (differences in the level of hormones, disease dynamics, level of carotenoids, etc., e.g., [56]) and sex may have also played some important role. Although 39 out of the 90 Elban samples investigated by Tanini and colleagues [22] were collected in the same three localities of the present study, regrettably, genetic and microbiome data were not obtained for the same individuals in the two studies. Altogether, these reasons can explain why the gut microbial communities of the two Elban subpopulations did not mirror host genetics (e.g., nuclear diversity estimates) for what concerns similarity in alpha diversity levels, even if they displayed very different community structures, thus reflecting the genetic divergence of the two *A. rufa* island demes.

Among many extrinsic and intrinsic factors [16], environment and host genetics are the main drivers shaping the gut microbiome composition in wild populations, with either of the two being prevalent according to different situations and organisms [40,45,57,58]. Several studies investigated the role played by host genetics, which appears to be stronger in mammals than in birds [59,60]. Nevertheless, most research focused on differences among distinct bird species [61,62], while microbiome shaping at a within-species level received much less attention so far. For example, Fleischer and collaborators [61] found little support for a large-scale control of the microbiome by host genetics, suggesting this would not necessarily imply the same at lower scales. In the present study, the correspondence we found between the spatial genetic and microbiome structure in the Elban partridges seems to point toward this direction, although further, specifically designed studies are needed to better investigate this issue.

The first investigation of the gut microbiome in wild *A. rufa* lays the foundations to improve some aspects in the management of this species. The Elban population—the only natural, long-established, and self-sustaining [22] of Italy—can work, indeed, as a reference in studies aimed at exploring the gut microbiome of *A. rufa*. A comparison with the most important farmed populations of Italy would be valuable for ex situ managers, for instance, to better understand the effect of the preventive supply of antimicrobials and coccidiostatics to captive birds until their release in nature [63]. Likewise, a comparison between the gut microbiome of native and introduced *A. rufa* should be pursued as well as the occurrence of red-legged partridges imported from abroad is a matter of fact across the species’ range [64]. In particular, we agree with Lavretsky and co-authors [65], who successfully reported over a heavily managed and worldwide translocated gamebird, the mallard (*Anas platyrhynchos*), that reciprocal interactions between natural and captive-bred individuals—we add, also those occurring at the microbial scale—will ever increasingly lead to the rise of admixed populations in light of ongoing rapid habitat transformation and climate changes. Indeed, as in the mallard, introduced *A. rufa* populations are capable to adapt locally even within a few decades since farm releases (e.g., [66]).

## 5. Conclusions

We investigated for the first time the microbiome of *A. rufa,* focusing on a wild protected island population, and we found that both its composition and genetic diversity varied at a small spatial scale. The divergence between the microbes associated with birds living on the opposite sides of Elba Island agreed with the known spatial genetic structure of the same *A. rufa* population. As we already suggested in this previous study, we advise the National Park that the two subpopulations should be managed separately also to avoid any loss and/or significant homogenization in the microbiome structure of the Elban population.

## Figures and Tables

**Table 1:** Sample information including name, locality, subpopulation, date, and elevation.

Sample	Locality	Subpopulation	Date	Elevation
SBART 13	San Bartolomeo	WEST	17 February 2019	402 m
SBART 14				
SBART 19				
SBART 20				
SBART 21				
SBART 22				
PM 26	Pietra Murata	WEST	8 February 2020	547 m
PM 41				
PM 43				
PM 44				
PM 45				
PM 46				
CDM 3A	Cima del Monte	EAST	15 December 2018	428 m
CDM 3B				
CDM 3C				
CDM 3MIX				
CDM 4A				
CDM 4B				

## Data Availability

Sequences (raw reads) were deposited in the European Nucleotide Archive (ENA) at EMBL-EBI under study accession number PRJEB59418.

## Supplementary Materials

The following supporting information can be downloaded at: https://www.mdpi.com/article/10.3390/ani13213341/s1, Table S1. Kruskal–Wallis tests for alpha diversity indexes among the three sampling sites (SBART, PM, and CDM) and the two subpopulations (WEST, EAST). Statistically significant comparisons are reported in bold (*p* < 0.05, see also Figure 3). Table S2. Permanova tests among the three sampling sites (SBART, PM, and CDM) and the two subpopulations (WEST, EAST) carried out with different metrics (999 permutations). Statistically significant comparisons are reported in bold (*p* < 0.05). Figure S1. Rarefaction curves of alpha diversity approaching the saturation plateau (sampling depth, 7701). Each sample with relative color is reported in the box to the right side. Presence and abundance of the genus *Faecalicatea* is indicated by an asterisk. Figure S2. Relative abundances of bacterial genera in the libraries as obtained for the three sampling sites. Each bar corresponds to one sample (single red-legged partridge).
