# Supplementary material for: Wild Avian Gut Microbiome at a Small Spatial Scale: A Study from a Mediterranean Island Population of Alectoris rufa
Source: Animals (Basel). 2023 Oct 27;13(21):3341. doi: 10.3390/ani13213341 (PMC10648672; doi:10.3390/ani13213341)

Supplementary Materials

Wild Avian Gut Microbiome at a Small Spatial Scale: A Study from a Mediterranean Island Population of *Alectoris rufa*

Monica Guerrini, Dalia Tanini, Claudia Vannini, Filippo Barbanera

Content

Table S1	Page 2
Table S2	Page 3
Figure S1	Page 4
Figure S2	Page 5

Table S1. Kruskal-Wallis tests for alpha diversity indexes among the three sampling sites (SBART, PM, and CDM) and the two subpopulations (WEST, EAST). Statistically significant comparisons are reported in bold ($p < 0.05$, see also Figure 3).

	ASVs	Shannon	Pielou's Evenness
	p	*p*	*p*
SBART-PM	0.584	0.749	0.749
SBART-CDM	**0.006**	**0.004**	**0.004**
PM-CDM	**0.004**	**0.004**	**0.004**
WEST-EAST	**0.003**	**0.001**	**0.001**

Table S2. Permanova tests among the three sampling sites (SBART, PM, and CDM) and the two subpopulations (WEST, EAST) carried out with different metrics (999 permutations). Statistically significant comparisons are reported in bold ($p < 0.05$).

	Jaccard	Bray-Curtis	unweighted Uni-Frac	weighted Uni-Frac
	p	*p*	*p*	*p*
SBART-PM	**0.011**	**0.001**	0.206	0.206
SBART-CDM	**0.004**	**0.004**	**0.024**	**0.002**
PM-CDM	**0.002**	**0.001**	**0.001**	**0.002**
WEST-EAST	**0.001**	**0.001**	**0.005**	**0.001**

Figure S1. Rarefaction curves of alpha diversity approaching the saturation plateau (sampling depth, 7701).

Each sample with relative color is reported in the box to the right side.

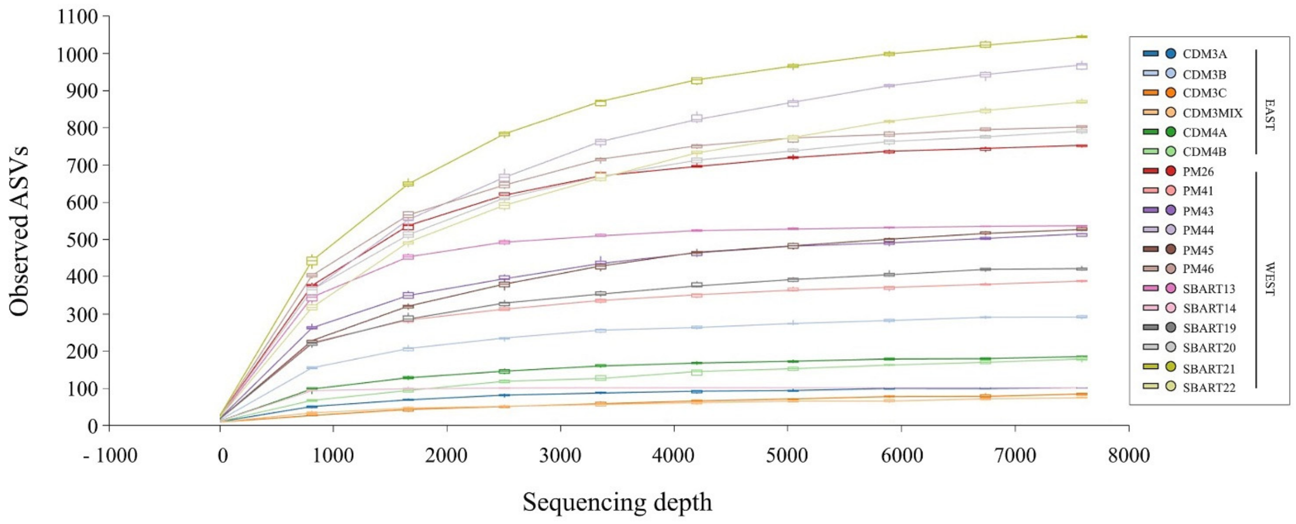

Figure S2. Relative abundances of bacterial genera in the libraries as obtained for the three sampling sites. Each bar corresponds to one sample (single red-legged partridge). Presence and abundance of the genus *Faecalicatea* is indicated by an asterisk.

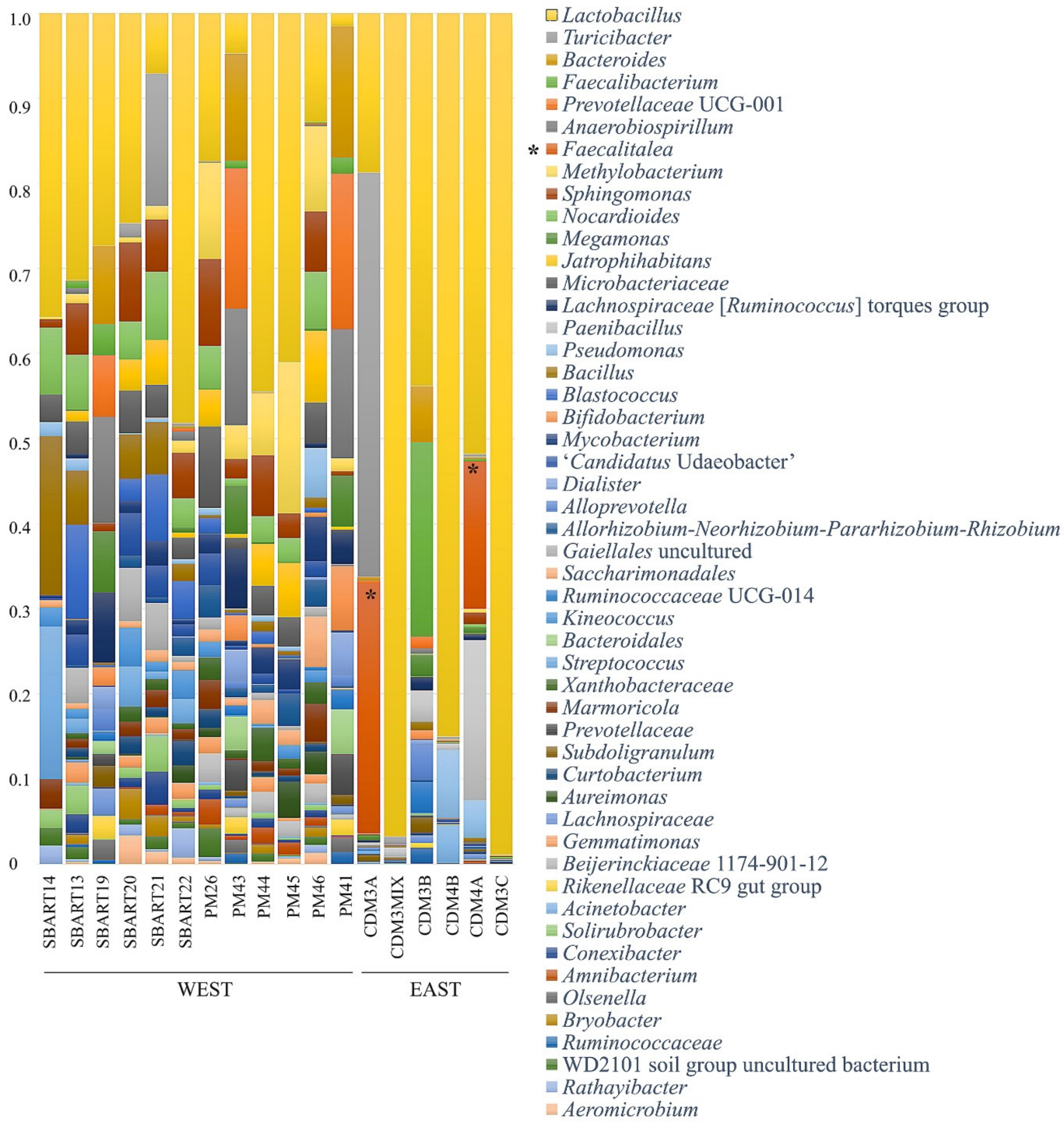

Supplement: Supplementary file 1 [file animals-13-03341-s001.zip › animals-2641062-supplementary.pdf]
